# Change in practice: a qualitative exploration of midwives’ and doctors’ views about the introduction of STan monitoring in an Australian hospital

**DOI:** 10.1186/s12913-018-2920-5

**Published:** 2018-02-17

**Authors:** M. E. Mayes, C. Wilkinson, S. Kuah, G. Matthews, D. Turnbull

**Affiliations:** School of Psychology, University of Adelaide, Room 721a, Hughes Building, North Tce, Adelaide, SA 5005 Australia

**Keywords:** ST-analysis, Foetal monitoring, Technology, Organisational change, Views of healthcare professionals

## Abstract

**Background:**

The present study examines the introduction of an innovation in intrapartum foetal monitoring practice in Australia. ST-Analysis (STan) is a technology that adds information to conventional fetal monitoring (cardiotocography) during labour, with the aim of reducing unnecessary obstetric intervention. Adoption of this technology has been controversial amongst obstetricians and midwives, particularly as its use necessitates a more invasive means of monitoring (a scalp clip), compared to external monitoring from cardiotocography alone. If adoption of this technology is going to be successful, then understanding staff opinions about the implementation of STan in an Australian setting is an important issue for maternity care providers and policy makers.

**Methods:**

Using a maximum variation purposive sampling method, 18 interviews were conducted with 10 midwives and 8 doctors from the Women’s and Children’s Hospital, South Australia to explore views about the introduction of the new technology. The data were analysed using Framework Analysis.

**Results:**

Midwives and doctors indicated four important areas of consideration when introducing STan: 1) philosophy of care; 2) the implementation process including training and education; 3) the existence of research evidence; and 4) attitudes towards the new technology. Views were expressed about the management of change process, the fit of the new technology within the current models of care, the need for ongoing training and the importance of having local evidence.

**Conclusions:**

These findings, coupled with the general literature about introducing innovation and change, can be used by other centres looking to introduce STan technology.

**Electronic supplementary material:**

The online version of this article (10.1186/s12913-018-2920-5) contains supplementary material, which is available to authorized users.

## Background

The Australian healthcare sector is currently undergoing change, facing ongoing demands from drives for reform [1]. Additionally, it faces significant challenges in coping with constantly changing technology [2]. From an international perspective, the recent amount of innovation and the commensurate impact on health care has been unprecedented in history, both clinically and economically [3]. Although medical teams are continuously being confronted with ongoing changes to the way they practice, it is important to acknowledge that implementing new technology into routine care can be a slow and unpredictable process [4].

Successful implementation of technology has been defined as the incorporation or routine use of such technology within an organisation [5, 6]. Failure to adopt new technologies is common, even when there is evidence of potential benefits to patients and to the overall health care system [7, 8]. Failure to adopt can come as a result of aspects of the physical environment such as the usability of the technology, or individual factors such as staff and patient opinions and attitudes. Understanding the views of staff can be particularly important when introducing new technology, as front-line users are critical for the success of the implementation [9]. Some of the key person-orientated factors identified as contributing to the uptake of technological innovations are: authority structures/senior management support [6]; attitudes and perceptions of key staff members [7]; consistencies of values in reference to clinical practice [10]; and knowledge transfer and training [11, 12].

Research shows that the failure to assess the human aspect of the implementation of technology can result in misuse, which has implications for patient safety [13]. Organisational issues such as miscommunication between staff and decreased ability to deviate from routine are two key issues. This may be due to the fact that implementation of new technology often requires experimentation, using trial and error to find solutions that work [14] and help-seeking [15]. Failed investments in technology can be costly and cause dissatisfaction among employees [16]. Therefore, staff views and opinions must be sought to understand the technology implementation process from an organisational perspective.

A recent innovation in the foetal monitoring industry is ST-analysis (STan) (introduced in Sweden in 2000 by Neoventa Medical), which is a method and devices that monitor the foetus during labour and as a concept has a strong focus on education and training. The existing standard of care in Australian hospitals includes the use of cardiotocography (CTG) monitoring of the foetus. CTG exclusively monitors the heart rate of the foetus and is most often monitored externally via telemetry [17]. CTG has a high false positive rate of over 50%, and if used alone can increase the chance of interventions, such as emergency caesarean sections and operative vaginal births, without any significant benefit to the perinatal outcome [17]. STan is recommended for use in high-risk pregnancies and aims to overcome the shortcomings of CTG by assessing the foetal heartbeat, in conjunction with the foetal response to reduced availability of oxygen (hypoxia) [17]. This is made possible by an internal monitoring instrument, a foetal scalp electrode (“scalp clip”). The scalp clip then assists in differentiating between a foetus exposed to hypoxia that is compensating well and a foetus that is unable to compensate according to the analysis of the ST interval in the ECG [17]. STan technology has been trialled and introduced into hospitals throughout Europe, the United Kingdom and the USA and has had inconsistent clinical results [18]. However, the caesarean section rates vary widely in these settings ranging from 16% in Norway through to 22% in the United Kingdom compared with 30% in Australia [19]. A research investigation into barriers to uptake of STan in Swedish, Norwegian and hospitals in the UK was conducted 13 years after the commencement of the randomised controlled trials. The study found the main barriers to uptake included low commitment in the implementation process and absence of opinion leaders. [20]. At the broader international level however, there been a failure to investigate organisational issues associated with the implementation of STan.

This aim of the current study is to explore the attitudes and concerns of midwives and doctors about the introduction of STan in an Australian context. The setting for the current study is the first hospital to introduce STan in Australia as a part of a trial^1^ [21]. It is hoped that this study will assist in developing an understanding of the contributing factors to the uptake of STan in Australia. It is anticipated that by gaining a more thorough understanding of care provider views, Australian hospitals that introduce this new technology in the future may be better able to manage the associated organisational change.

## Method

### Context

The study was conducted at the Women’s and Children’s Hospital in Adelaide, South Australia, a high risk specialist facility with approximately 5000 deliveries per annum. In this setting high risk pregnancies are predominantly monitored using CTG and foetal scalp samples. The study was undertaken in the context of a pilot randomised control trial [21]. The premise for the trial was that the existing mixed findings about STan were not necessarily generalisable to Australia due to variations in clinical practice. Prior to the commencement of the trial an independent international expert in CTG and STan conducted staff training. All midwives and doctors working in the labour ward were also required to undertake a minimum of 2 h of training by “super-users”, of which there were 3 doctors and 12 midwives, followed by compulsory assessment. As preparation for introduction of STan monitoring in 2015, all 138 clinicians (100%) were trained and certified (40 medical, 98 midwives).

### Participants and recruitment

A maximum variation purposive sampling method was used to produce a sample broadly representative of the population of interest [22–24]. We aimed to interview staff with a range of clinical experiences based on years of service at the hospital and roles in various clinical areas. Participants ranged in age between 24 and 58 and included eight doctors and ten midwives.

### Data collection

Data were collected via audiotaped semi-structured one-to-one interviews at the hospital between June 2015 and August 2015. This corresponded to the time that the pilot trial was commencing although STan had already been introduced into the hospital 7 months prior to this period. The interview approach is appropriate for this qualitative study as it provides flexibility and depth in acquired information and allows the participants to fully explain their views and experiences [25]. The first author who was not associated with the hospital and who had no vested interests conducted all interviews. Thus the participants were able to establish rapport and trust in the researcher by understanding that their honest opinions would be kept anonymous and independent of hospital administration.

The interview schedule consisted of ten questions that first asked about the participants’ opinions of the use of STan, followed by more general questions concerning health care innovation and the organisational change process (Additional file 1). In order to ensure consistency across interviews, the interviewer followed the question guide closely with the intention of not influencing participant responses [26].

The average duration of the interviews was 13 min. The nature of a busy working hospital resulted in some interviews being restricted in terms of duration. Some midwives and doctors had little time while they were on shift to discuss their answers in depth with the interviewer, however a number of initial interviews were lengthy. These interviews provided in- depth information, which was often repeated in later interviews in less depth by subsequent participants. After 12 interviews, the data were initially analysed by the researcher and *respondent validation* was sought [27] via a meeting that included some staff who were participants in the study. Feedback indicated a high level of face validity in the findings. Interviews continued until data saturation was achieved at around participant 15; however, continuation of data collection occurred until participant 18, to ensure that all clinical roles were appropriately represented [27]. The final findings of the study were presented to staff again to re-check for face validity.

### Data analysis

The initial three interviews were transcribed verbatim by the first author to gain familiarity with the data [28] and the subsequent 15 audio files were sent to a professional transcription service where they were also transcribed verbatim. A coding list was developed guided by the objectives of the study and the themes developed as a consequence of the face validity testing process for the initial data. To ensure *interpretive rigour* [29], two additional researchers reviewed the themes and codes and adjustments were made. In addition, an audit trail was kept through the process of data collection to ensure transparency and to assist with later analysis [30].

#### Framework analysis

Data were analysed via Framework Analysis, a form of thematic analysis, widely used in applied health research, which produces highly structured output data that allows for comprehensive and systematic data analysis [31]. The following 7-step process was used: *1. Transcription. 2. Familiarisation with the transcript. 3. Coding. 4. Developing an analytical framework. 5. Applying the analytical framework to the raw data. 6. Charting data into the framework matrix. 7. Interpreting the data* [32].

## Results

The final data consisted of 18 interviews with 8 doctors and 10 midwives (Table 1).

**Table 1:** Participant characteristics

Profession	Mean Age (range)	Gender	Team areas represented	Mean years of service
10 Midwives	53 (45–58)	10 females	Midwifery Group Practice; Pregnancy Induction Assessment Suite; Delivery Suite	17
8 Doctors	33 (24–44)	2 males 6 females	Obstetrics and Gynaecology; Women’s and Baby’s division.	3^a^

^a^One participant did not report their years of service

Four major themes that describe the opinions and perceptions of staff were identified:

- Philosophy of care;
- Implementation process including education and training;
- Research evidence;
- Attitudes toward the STan technology

In the presentation of results, the interviewees are referred to with their interview number and professional group membership.

### Philosophy of care

A number of factors in relation to staff values and philosophies of care for labouring women were expressed as reasons for reservation about Stan. Participants, most notably the midwives, reported that the introduction of STan monitoring would lead to changes in the current model of care, with it becoming more interventional.

*I can also see it maybe being a bit more interventional, 'cause women have to have their membranes ruptured, and have to have the scalp clip on the baby's head, so just those simple things can be interventional. (Interview 1: MW, [Midwife])*

There was also the perception that STan would create “abnormal” care for laboring women, quite different to a “normal” birthing experience where there is minimal intervention.

*…“cause you'd need an epidural and there... to be able to cope with labour… you know the technology is good, but you'd have to have an epidural, then you just... I don't know it's very ‘medicalised’”. (Interview 5: MW)*

In addition to this perceived change in the model of care, many participants made reference to the lack of telemetry with STan (the model bought by the organisation), which allows women to be mobile during labour. It was expressed that this would lead to women being “tied to the bed” (interview 5, MW), resulting in a more lengthy and uncomfortable labour, as well as not being able to get in the bath.

*…it will stop her from being able to be mobile, and therefore affect the progress of her labour. So in other words, it's counter-productive. (Interview 7: MW)*

Participants also emphasised the importance of taking the preferences of laboring women into account, coupled with a general concern about the extent of intervention for the women and their babies.

*And then patient preference as well, so we've got to talk to them about what they want, whether they want to be mobile…I think one of the reservations I have is having to put a scalp electrode on every baby. And I know that this feedback we're getting from some of the women and couples in labour are that they don't really like that. (Interview 2: DOC)*

### Implementation process, including education and training

Common comments about the implementation of STan were to do with the way in which it was introduced into practice, including issues such as the education and training of staff, timing and approach of the introduction, and the influence of individuals within the organisation.

Interviews revealed diversity in experiences with the education and training program. Some reported it to be a “very rushed session…” (Interview 5 MW); in contrast others commented: “we've all been offered plenty of opportunity for training, we've been offered lots of support…” (Interview 1: MW). Some participants indicated feeling “overwhelmed, as there was not adequate time to prepare for both the information overload and the exam following.” (Interview 17: MW)

*…there was enormous unnecessary pressure, um at that time when the initial information was handed out, with a test involved that we were told we must pass… at the initial start I think that created a lot of tension and a lot of um a bit of a barrier for some people to think that this is a good thing… (Interview 17: MW)*

Overall, the comments about training indicated that there was need for ongoing support and practical training on the ward.

*…education is going to be a big part of it, making sure we all understand how to use it, how it works, what the limitations are, the benefits are, before we actually have to use it in our day-to-day practice. (Interview 2: DOC, (Doctor))*

Participants also reported receiving conflicting information by their national professional body at the time STan was introduced, which resulted in staff finding it “really confusing” (Interview 10, DOC).

*It was rolled out at the same time that we did the FSCP [Foetal Scalp Clip Protocol], which was from the RANZCOG guidelines for foetal monitoring and they're slightly different. So that added to some of the confusion. So I think it was a bit of a timing issue.(Interview 5: MW)*

In addition, participants described a delay between education and training about STan to it being introduced onto the ward. There was widespread belief that this created a lack of confidence in staff using the technology and resulted in some anxiety and frustration when it came to using it on the ward.

*…the actual sort of teaching and learning, theoretical learnings were a fair long way away from the actual physical introduction of the technology. So I think you do find that you've forgotten pretty much most of it by the time. (Interview 4: MW)*

There were also reservations about the way STan was introduced by the organisation, with one participant describing it feeling very “sudden” (Interview 14: MW). Participants expressed the need for a more collaborative approach in the early stages to increase awareness and a sense of ownership in practice over the change.

*…in general when there's change introduced, whether it's technology or not, one of the most important things is talking to the people it's going to affect before it's introduced. (Interview 2: DOC)*

“Internal influencers” were observed as being a key barrier to the uptake of STan. This was described as the lack of endorsement by key staff members, together with the hierarchical nature of the hospital environment.

*…people had a lot of different feelings about it. Like some people thought it wasn't really useful, other people were really, really for STan, and then the whole bunch of other people kind of like in the middle… STan doesn't really get mentioned unless certain people are around. (Interview 3: MW)*

As a result of difficulties with training and the limited opportunities to use the technology, participants expressed the desire to see more results from using STan. One participant commented: “I've just got to actually see the technology being used more to work out what I think about it” (Interview 15: DOC).

*I've had pretty minimal experience… we're still not using it very often I've got to say… (Interview 2: DOC)*

Research evidence

The interviews revealed a range of sometimes conflicting views about the research evidence. Perceptions about lack of clinical evidence about the benefits of STan, was a prominent barrier to uptake for many participants, in particular doctors.

*I think if there's substantial evidence behind the change in practice people are more likely to support it. (Interview 15: DOC).*

At the same time, participants expressed the belief that existing international evidence on the effectiveness of STan would not be relevant for the Australian population.

*…because it [STan] hasn't been used in our patient population, which is pretty different to the patient populations it's been used on in Europe. We don't have any local data at all. And the way we manage our women and how high risk some of our women are is different to the patient populations that they've studied as well… (Interview 2: DOC)*

Many participants also agreed that conducting the trial at the hospital was a good thing because the data would be relevant to their patient population.

*I think it's good that we're doing a study, certainly a trial here…rather than basing our data on things that are done in other countries, or other states. (Interview 13: DOC)*

Other concerns were about the concurrent routine implementation of the technology and the research trial. Participants expressed feelings of discomfort and emphasised the need for the trial to be completed first.

*I feel a little bit uncomfortable about it. I would like the trial done before we used it routinely. (Interview 15: DOC)*

Many participants reported reservations about using the technology in the absence of endorsement by professional bodies.

*I guess I would be a bit wary about the fact that our College doesn't support STan at this stage…and the RANZCOG foetal monitoring guidelines are different to I guess what we've been using. (Interview 13: DOC)*

Participants expressed anxiety about the reliance on the technology to produce an electronic alarm prior to decisions to go to operative delivery. Some believed this to be difficult for those who have to make the final decision in instances that the output of the monitor was at odds with prior clinical experience of monitoring with CTG alone.

*I think it's more concerning for people who've been working in obstetrics for longer, and seeing CTGs they would have normally taken to section, and not being able to do that now. Or sitting on them for longer is... makes them a little bit nervous. (Interview 15: DOC)*

Alternatively, some participants reported that they believed STan actually creates a sense of ease, as the decision to go to operative birth is assisted by the technology and that removing the opinions of individuals can be a positive thing.

*Definitely examples that I've seen anecdotally on the labour ward where people [women in labour] have not been taken to section 'cause people [clinicians] are more comfortable to sit on traces we would have normally sectioned. (Interview 15: DOC)*

### Attitudes towards the STan technology

The final theme that emerged was concerned with the added benefits of STan, feelings of distrust toward the technology and operational issues with respect to the machine itself. One participant suggested that “it helps us to interpret CTGs where they're not clear cut” (Interview 2: DOC). This would suggest that there are levels of trust in the technology, with it being able to provide further information that is needed to assist the decision making process. In contrast, some participants felt that the move away from clinical judgement and the reliance on the machine was concerning, reporting that there was a need for appropriate and correct interpretation.

*I think it also needs to be used appropriately, but also interpreted appropriately too, because we're going to be watching this screen a lot more, and looking at this trace... (Interview 1 MW)*

Some participants suggested that they understood the technology would alarm when necessary, but that high quality education about interpreting the alarm signals was needed in order for clinicians to be confident about decisions to proceed or not, to caesarean section. Others commented on the need to “all believe in the same technology” (Interview 8: MW) and that this would help team cohesion and acceptance of STan throughout the ward.

*“I don't want to be making big decisions at this stage without consulting someone who's more used to using the technology. So, yeah, so that doesn't... that's not always available. So I think that's one of the barriers to picking it up, if there's no-one who's more experienced or more confident whether around, then perhaps they don't want to start it in the first place” (Interview 12: DOC).*

‘Operational factors’, associated with the technology, was one of the most widely reported barriers to using STan. One participant commented on STan as being “sort of a bit sub-standard” (Interview 5: MW), while others reported only using the machines as CTG monitors, which is in line with their current practice.

*But we haven't actually switched them over to be STan enabled. We've just used them purely as a foetal monitor like we would use a (brand name) one that we've got. (Interview 1: MW)*

The requirement to apply the scalp clip at the outset irrespective of the clinical situation was also a barrier for some.

*I guess it's the fact that you have to actually put in the scalp clip on to like any woman that you're considering, and you have to do it before you know anything's happened for it to be effective. (Interview 3: MW)*

## Discussion

The current study investigated midwives’ and doctors’ views about the introduction of STan foetal monitoring and is the first study of its kind in an Australian setting. STan monitoring is not used in any other centre in Australia and is yet to be recommended by the professional bodies because of a lack of evidence that it is beneficial in an Australian setting. At the time of the trial and current study the existing evidence base was not necessarily generalizable to Australia, as the Australian emergency caesarean section rate is significantly higher than other settings for STan trials [18, 19, 33, 34],.^2^ Should STan technology prove clinically efficacious in an Australian setting, this research will facilitate its successful its introduction, including the implementation of training and education. STan generally represents a change in practice from the method of using CTG for feotal monitoring, which has been clinical practice for over 40 years [17]. The discussion of the findings will be presented from the perspective of introducing organisational change.

The findings of this study suggest that some resistance to STan currently exists at the study hospital. It reveals a number of cognitive and interpersonal barriers to uptake. Cognitive factors consider the way participants think about technology, including operational factors and their attitudes towards innovations in general [35]. Interpersonal factors, such as the impact of key individuals on the change management were also found [35]. Additionally, education and training was noted to be a significant factor related to the implementation of STan.

The current study offers important insight into how healthcare professionals respond to the introduction and implementation of new technology. Research has found a number of barriers to the uptake of STan in Swedish hospitals [19]. The current study found a number of similar barriers exist for doctors and midwives in an Australian setting, including those related to philosophy of care; training and education and attitudes toward the technology. The present research reveals two other important aspects, namely the implementation process and perceptions about the research evidence and its fit with clinical knowledge.

STan foetal monitoring was perceived to be incongruent with the current philosophy of care. The findings suggest participants believed that the introduction of STan would increase interventions and result in a more “medicalised” process of labour, which many midwives and some doctors believe to be an undesirable outcome. This view is consistent with previous findings in the organisational culture and change literature, which indicates that resistance is significantly influenced by the values of the people within an organisation and specifically a failure to align the change with these values [11]. This may have been more pronounced in the current study as a majority of the midwives had been a part of the organisation for on average 17 years and therefore will strongly align with the culture and values present [11]..

This lack of alignment with the values of the individuals and teams involved with the move to use STan technology was broadly associated with participants’ attitudes toward the technology. Participants’ discussions about the use of STan were framed by perceptions of difficulty in the interpretation of the STan traces as well as the need for more experienced users. This aligns with the literature about technological innovation, which indicates that the perceived ease of use of technology has significant positive influence on actual usage and uptake of innovation [36].

Further, Attewell [12] emphasises the role of organisational learning, skill development, and a reduced knowledge base as potential barriers to adoption of innovation, and found that as knowledge barriers are lowered, the spread of innovation gains momentum [12]. According to Edmondson, Bohmer and Pisano [35], successful implementation includes a four-stage process including: individual enrolment to motivate the team, preparatory practice sessions and early trials within the organization, and promoted process improvement through reflective practices [35].

As health care organisations often rely on hierarchical structures, individual influencers have the ability to affect the implementation process [37]. This is demonstrated in the current study and is present in the literature of learning behaviour in the workplace, where “psychological safety” [8] is a concern for employees. Psychological safety impacts on an individual’s ability to feel comfortable openly making and learning from mistakes [8]. Participants discussed the anxiety observed in colleagues who had to make key decisions about acting on STan traces, which may also be impacted on by the organisational culture of the hospital. Authority structures can promote or inhibit collective learning in a number of ways. Individuals in positions of authority, such as project and team leaders, may influence the technology learning process by coordinating the activities in an implementation project. Furthermore, people are highly aware of the behaviour of those in positions of authority [38] and dependent on them for recognition and preferred assignments [39, 40].

The results also reveal that the lack of professional body endorsement of STan was associated with discomfort in the routine use of the technology. The status of the research evidence was an important issue within the participant population, and this can relate to psychological safety [8] in the profession. This was further complicated by the presence of contrasting expert knowledge regarding foetal monitoring as well as varying comfort levels of experienced practitioners with different monitoring methods [41].

The study must be considered within its limitations. It was conducted as a “snapshot” in time, representing only initial responses to the implementation of STan. In addition, the interviews took place at one Australian site, which may not be translatable to other hospitals around Australia. However, the sample represented a number of areas and roles across the maternity ward and a wide range of views about STan were obtained. Findings from the current study may be relevant to other Australian hospitals preparing for the implementation of STan monitoring. Moreover, the general literature about barriers to the implementation of new technology may assist in planning for and education about STan monitoring.

## Conclusion

This study explored the views of healthcare professionals in relation to the introduction of STan for feotal monitoring during labour. We found that the views were influenced by four main factors: alignment with the hospital’s philosophy of care, training and the process of the introduction to STan, the impact of current available research evidence, and the general attitudes to the new technology. Participants made suggestions for increased and ongoing education and the need for a collaborative implementation process, to improve the understanding and uptake of STan within the organisation. It is the suggestion of the authors that this study be repeated after the results from the randomised control trial are released.

## Additional file

Additional file 1 Interview schedule. All questions included in the qualitative interviews. (DOC 41 kb)

## Abbreviations

CTG: Cardiotocography
DOC: Doctor
MW: Midwife
RANZCOG: Royal Australia New Zealand College of Gynaecologists
STan: ST analysis
WCH: Women’s and Children’s Hospital

## Footnotes

1 At the time of the study, STan was not supported in Australian guidelines.

2 After the current study was completed an RCT was published regarding the US population who have a similarly high caesarean section rate of 30% [19, 33]. However, the two settings are not directly comparable as the model of care differs with the US having minimal Midwife driven care [34].The STan method was found to produce insignificant differences in caesarean section outcomes compared with standard CTG [34].
