# Supplementary material for: Change in practice: a qualitative exploration of midwives’ and doctors’ views about the introduction of STan monitoring in an Australian hospital
Source: BMC Health Serv Res. 2018 Feb 17;18:119. doi: 10.1186/s12913-018-2920-5 (PMC5816378; doi:10.1186/s12913-018-2920-5)
Supplement: Supplementary file 1 — Interview schedule. All questions included in the qualitative interviews. (DOC 41 kb) [file 12913_2018_2920_MOESM1_ESM.doc]

**INTERVIEW SCHEDULE**

*Interviewer: “These questions aim to discover more about your experience and your opinions related to the use of ST-an technology. Please be honest in your responses as a wide range of opinions is necessary to inform our research”.*

1. What is your understanding of ST-an technology?
2. How much exposure have you had to the Neoventa machine and also using the ST-analysis function?
3. What **concerns or reservations**, if any, do you have about the use of this technology?
4. What do YOU believe are the **benefits** of using ST-an technology, first, for your team and then for the women and their babies?
5. What factors would you consider when deciding whether to use ST-analysis?
6. How do you feel about using ST-analysis at this stage?

6.b) in practice today, would you use St-an on a woman if she met the criteria?

*Interviewer: “The medical field is a rapidly changing environment with new information and new technologies being introduced at a rapid rate”.*

1. Think about a previous experience, whether in your current role or a previous role, where a new way of doing things was **successfully** introduced. What do you believe were the reasons for its successful implementation?
2. Now, think about a time where a new way of doing things was **unsuccessfully** introduced. What do you believe were the reasons for the outcome?
3. How do you think the introduction of ST-an could be improved?
4. What advice would you give to administration at ANOTHER Australian hospital on the implementation of ST-an?

*Interviewer: “Do you have anything else you would like to share?”*
